# Frequency of the *STRC*-*CATSPER2* deletion in *STRC*-associated hearing loss patients

**DOI:** 10.1038/s41598-021-04688-5

**Published:** 2022-01-12

**Authors:** Shin-ya Nishio, Shin-ichi Usami

**Affiliations:** Department of Hearing Implant Sciences, Shinshu University School of Medicine, 3-1-1 Asahi, Matsumoto, 390-8621 Japan

**Keywords:** Genetics, Medical genetics

## Abstract

The *STRC* gene, located on chromosome 15q15.3, is one of the genetic causes of autosomal recessive mild-to-moderate sensorineural hearing loss. One of the unique characteristics of *STRC*-associated hearing loss is the high prevalence of long deletions or copy number variations observed on chromosome 15q15.3. Further, the deletion of chromosome 15q15.3 from *STRC* to *CATSPER2* is also known to be a genetic cause of deafness infertility syndrome (DIS), which is associated with not only hearing loss but also male infertility, as *CATSPER2* plays crucial roles in sperm motility. Thus, information regarding the deletion range for each patient is important to the provision of appropriate genetic counselling for hearing loss and male infertility. In the present study, we performed next-generation sequencing (NGS) analysis for 9956 Japanese hearing loss patients and analyzed copy number variations in the *STRC* gene based on NGS read depth data. In addition, we performed Multiplex Ligation-dependent Probe Amplification analysis to determine the deletion range including the *PPIP5K1*, *CKMT1B*, *STRC* and *CATSPER2* genomic region to estimate the prevalence of the *STRC*-*CATSPER* deletion, which is causative for DIS among the *STRC*-associated hearing loss patients. As a result, we identified 276 cases with *STRC*-associated hearing loss. The prevalence of *STRC*-associated hearing loss in Japanese hearing loss patients was 2.77% (276/9956). In addition, 77.1% of cases with *STRC* homozygous deletions carried a two copy loss of the entire *CKMT1B-STRC-CATSPER2* gene region. This information will be useful for the provision of more appropriate genetic counselling regarding hearing loss and male infertility for the patients with a *STRC* deletion.

## Introduction

Congenital hearing loss is one of the most common sensory disorders, occurring in 1 in 700–1000 newborns, with approximately 60% of cases attributable to genetic causes^1^. The *STRC* gene, which is located on chromosome 15q15.3 in what is known as the DFNB16 locus, is one of the genetic causes of autosomal recessive mild-to-moderate sensorineural hearing loss^2,3^.

One of the unique characteristics of *STRC*-associated hearing loss is the high prevalence of long deletions or copy number variations observed on chromosome 15q15.3. Chromosome 15q15.3 contains segmental duplications including five functional genes and three pseudo-genes, *PPIP5K1* (MIM: 610,979), *CKMT1B* (MIM: 123,290), *STRC* (MIM: 606,440), *CATSPER2* (MIM: 607,249), *PPIP5K1P1*, *CKMT1A* (MIM: 613,415), *STRCP1*, and *CATSPER2P1*. Pseudo-*STRC* (*i.e., STRCP1*) is located downstream of the *STRC* gene and shows 98% homology to the functional *STRC* gene. This high degree of homology makes variant interpretation in the analysis of next-generation sequencing results challenging. *STRC*-associated hearing loss is a relatively common genetic cause of non-syndromic hearing loss, with an incidence of 5.4–16.1% in hearing loss populations with mixed ethnicity^4,5^, 6–11.2% in American hearing loss patients^6,7^, 6% in Italian hearing loss patients^8^, 1.7–2.4% in Japanese hearing loss patients^9,10^, 2.6% in Turkish autosomal recessive hearing loss patients^11^, 10% in Czech mild-to-moderate hearing loss patients^12^, 10.8% in Korean mild-to-moderate hearing loss patients^13^, and 6% in *GJB2*-negative Germany hearing loss patients^14^.

Further, the deletion of chromosome 15q15.3 from *STRC* to *CATSPER2*, is also known to be a genetic cause of deafness infertility syndrome (DIS: OMIM 611,102), which is associated with not only hearing loss but also male infertility, as *CATSPER2* plays crucial roles in sperm motility^15,16^. Thus, data regarding the deletion range for each patient is important for appropriate genetic counseling in terms of hearing loss and male infertility. Several patterns regarding the deletion range of chromosome 15q15.3 have been reported (HGMD professional), with some patients harboring a deletion lacking only the *STRC* gene with the *CATSPER2* gene intact. Thus, the prevalence of the *STRC*-*CATSPER* deletion among *STRC*-associated hearing loss patients is thought to be valuable information.

In the present study, we performed next-generation sequencing (NGS) analysis for a large number of Japanese hearing loss patients (*n* = 9956) and analyzed copy number variations (CNVs) in the *STRC* gene using NGS read depth data. In addition, we performed Multiplex Ligation-dependent Probe Amplification (MLPA) analysis to determine the deletion range including the *PPIP5K1*, *CKMT1B*, *STRC* and *CATSPER2* genomic regions to estimate the prevalence of the *STRC*-*CATSPER* deletion, which is causative for DIS among *STRC*-associated hearing loss patients.

## Methods

### Subjects

We performed target re-sequencing analysis for 9956 unrelated Japanese sensorineural hearing loss patients (2069 autosomal dominant or mitochondrial inheritance cases, 5831 autosomal recessive inheritance or sporadic cases, 1944 unknown family history cases and 112 unilateral hearing loss cases) enrolled in this study from 90 otorhinolaryngology departments nationwide. Peripheral blood samples were obtained and DNA was extracted using a DNeasy blood and tissue kit (QIAGEN, Hilden, Germany). Evaluation of HL was performed by pure-tone audiometry for patients aged 4 years or older. The pure-tone average (PTA) was calculated from the audiometric thresholds at four frequencies (500, 1000, 2000 and 4000 Hz). The severity of HL was classified into 4 categories: mild (PTA 20-40 dB), moderate (41-70 dB), severe (71-90 dB) and profound (> 91 dB). The average age of participants was 26.5 (range 0 to 105), with 3812 males and 4819 females and 1325 without gender information.

Informed written consent was obtained from all subjects (or guardians in the case of minors) prior to participation. This study was approved by the Shinshu University Ethics Committee (Approval no.: 576) and the ethics committees of all other participating institutions. All procedures were performed in accordance with the Guidelines for Genetic Tests and Diagnoses in Medical Practice of the Japanese Association of Medical Sciences and the Declaration of Helsinki.

### Next-generation sequencing

Next-generation sequencing was performed for the 63 genes reported to cause non-syndromic hearing loss as described in a previous report^17^. In brief, amplicon libraries were prepared by using the Ion AmpliSeq Custom Panel, with the Ion AmpliSeq Library Kit 2.0 and the Ion Xpress Barcode Adapter 1–96 Kit (Life Technologies) according to the manufacturer’s instructions. After amplicon library preparation, equal amounts of the libraries for forty-five patients per one sequence run were pooled. After library preparation, next-generation sequencing analysis was performed using the Ion Proton system with an Ion P1 chip or the Ion S5 system with an Ion 540 chip according to the manufacturer’s instructions. We performed about 230 runs for the sequencing of 9956 patients. The averaged number of reads per sample was 1,684,289 reads (range 275,889–24,777,837) and the average depth of coverage was 667 (range 106–10,126).

The sequence data were aligned to the human reference genome sequence (build GRCh37/hg19) using the Torrent Mapping Alignment Program (TMAP). Subsequently, DNA variants were piled up with the Torrent Variant Caller plug-in software included in the Torrent Suit (Life Technologies).

The effects of the variants were analyzed by using ANNOVAR software^18^. The missense, nonsense, insertion/deletion, and splicing variants were selected from among the identified variants. Variants were further filtered to retain only those with a minor allele frequency under 1% in several control databases including the 1000 genome database (http://www.1000genomes.org/), the 6500 exome variants (http://evs.gs.washington.edu/EVS/), The Genome Aggregation Database (https://gnomad.broadinstitute.org), the human genetic variation database (dataset for 1208 Japanese exome variants) (http://www.genome.med.kyoti-u.ac.jp/SnpDB/index.html), the 8300 Japanese genome variation database (https://jmorp.megabank.tohoku.ac.jp/202102/) and the 333 in-house Japanese normal hearing controls. All filtering procedures were performed using our previously described original database software^19^. The pathogenicity of the identified variants was evaluated in accordance with the American College of Medical Genetics (ACMG) standards and guidelines^20^ with the ClinGen hearing loss clinical domain working group expert specification^21^. Copy number variant (CNV) analysis was performed according to our previous report^22^.

### MLPA analysis

MLPA analysis was performed to confirm the CNVs identified from the read depth data obtained by next-generation sequencing analysis. MLPA was performed using SALSA MLPA Probe mix P461-A1 and the SALSA MLPA EK1 reagent kit FAM according to the manufacturer’s instructions (MRC-Holland, Amsterdam, Netherlands). MLPA amplicon fragment lengths were analyzed by ABI 3130xl Genetic Analyzer (Applied Biosystems, Foster City, USA) with the GeneScan 500 LIZ Size Standard (Applied Biosystems, Foster City, USA). The fragment data were analyzed using Coffalyser.net software (MRC-Holland). We performed two duplicate tests for each sample.

## Results

### Prevalence and clinical features of *STRC*-associated hearing loss

In this study, we performed large-cohort next-generation sequencing analysis and CNV analysis for 9956 Japanese hearing loss patients. As a result, we identified 1258 (12.6%) patients carrying CNVs in 63 previously reported deafness genes. The most prevalent CNVs identified in this study were those in the *STRC* gene, which were observed in 1093 cases (231 cases carrying a 2 copy number loss, 612 cases carrying a 1 copy number loss, 243 cases carrying a 1 copy number gain and 7 cases carrying a 2 copy number gain). Among the 612 cases with a 1 copy number loss of the *STRC* gene, 41 cases carried single nucleotide variants (SNVs) or small insertion/deletion variants in the *trans* allele configuration. In addition to the 41 cases with SNVs or small ins/dels in the *trans* allele with the single copy loss of *STRC*, 1 case had compound heterozygous SNVs and 3 cases had homozygous SNVs. The SNVs or small insertion/deletion variants identified in this study are summarized in Table 1. Thus, a total of 276 cases were identified with *STRC*-associated hearing loss in this study, with the prevalence in Japanese hearing loss patients being 2.77% (276/9956) among all patients, 3.29% (192/5831) among autosomal recessive or sporadic hearing loss cases and 0.87% (18/2069) among autosomal dominant hearing loss cases. The *STRC*-associated hearing loss identified from autosomal dominant families is caused by pseudodominance as we previously reported^9^. When we restricted the patients to those with mild-to-moderate hearing loss, the prevalence of *STRC*-associated hearing loss was increased to 4.27% (132/3088). In addition, we identified only 2 cases with *STRC*-associated hearing loss among severe-to-profound hearing loss patients (0.07%; 2/2948). These two cases carried both a homozygous *STRC* deletion and a biallelic mutation of the *GJB2* gene. The severe-to-profound hearing loss observed in these two cases reflects the clinical phenotype of *GJB2*-associated hearing loss. This observed overlapping of both of the *GJB2* and *STRC* biallelic mutation is thought to result from the high carrier frequency of both of the *GJB2* mutations and the *STRC* deletion.

**Table 1:** SNVs or small insertion/deletion variants identified in this study.

Genomic coordinates (hg19)	dbSNP	Base change	Protein change	Allele number	SIFT	PP2	REVEL	CADD	gnomAD	8.3 K JPN	Pathogenicity
chr15:g.43903169A > T	–	c.3320 T > A	p.V1107D	1	D	B	0.428	21.3	0	0	VUS
chr15:g.43900289G > A	rs727503444	c.3670C > T	p.R1224X	2	–	–	–	41	0.000056	0	Pathogenic
chr15:g.43897469 T > G	–	c.3923A > C	p.Q1308P	2	D	P	0.382	17.3	0	0.0001	VUS
chr15:g.43897010A > G	rs756526028	c.3965 T > C	p.L1322P	1	D	P	0.785	26.2	0.000004	0.0001	VUS
chr15:g.43896218G > A	rs778909195	c.4351C > T	p.R1451X	1	–	–	–	37	0.000008	0	Likely Pathogenic
chr15:g.43895513C > G	–	c.4472G > C	p.C1491S	1	D	P	0.617	26.7	0	0	VUS
chr15:g.43895467_43895468delinsTG	–	c.4517_4518delinsCA	p.L1506P	2	–	–	–	–	0	0	VUS
chr15:g.43895466G > A	rs747194679	c.4519C > T	p.R1507W	1	D	B	0.437	23.2	0.000028	0	VUS
chr15:g.43893745_43893746del	–	c.4549_4550del	p.W1517Gfs*9	5	–	–	–	–	0	0	Pathogenic
chr15:g.43893746delA	–	c.4549delT	p.W1517Gfs*19	9	–	–	–	–	0	0.0007	Pathogenic
chr15:g.43893743C > T	–	c.4552G > A	p.G1518S	11	D	D	0.531	27.0	0	0.0005	Likely Pathogenic
chr15:g.43893742C > A	–	c.4553G > T	p.G1518V	3	D	D	0.61	32	0	0	VUS
chr15:g.43893725G > T	–	c.4570C > A	p.R1524S	1	T	B	0.35	22.6	0	0.0006	VUS
chr15:g.43893149C > A	rs147963245	c.4765G > T	p.V1589F	1	T	B	0.096	23.6	0.000004	0	VUS
chr15:g.43893136G > A	rs754953738	c.4778C > T	p.A1593V	1	D	D	0.625	32	0.000020	0	VUS
chr15:g.43892831G > A	–	c.4894C > T	p.Q1632X	1	–	–	–	37	0	0	Likely Pathogenic
chr15:g.43892277delC	–	c.5120delG	p.S1707Ifs*18	2	–	–	–	–	0	0	Likely Pathogenic
chr15:g.43892240C > A	–	c.5157G > T	p.E1719D	1	T	B	0.334	22.5	0	0	VUS
chr15:g.43892212G > A	rs750132696	c.5185C > T	p.R1729X	1	–	–	–	37	0.000012	0.0001	Likely Pathogenic
chr15:g.43892209G > A	rs139956283	c.5188C > T	p.R1730X	1	–	–	–	40	0.000025	0	Likely Pathogenic
chr15:g.43892178 T > C	–	c.5219A > G	p.K1740R	1	T	B	0.107	22.6	0	0.0001	VUS

gnomAD: allele frequency in the gnomAD database (https://gnomad.broadinstitute.org), 8.3 K JPN: allele frequency in the 8380 Japanese control population (https://jmorp.megabank.tohoku.ac.jp/202109/), PP2: PolyPhen2, T: tolerated, B: benign, D: deleterious or probably damaging P: possibly damaging, VUS: variant of uncertain significance. All variants are indicated on NM_153700.

**Figure 1:**
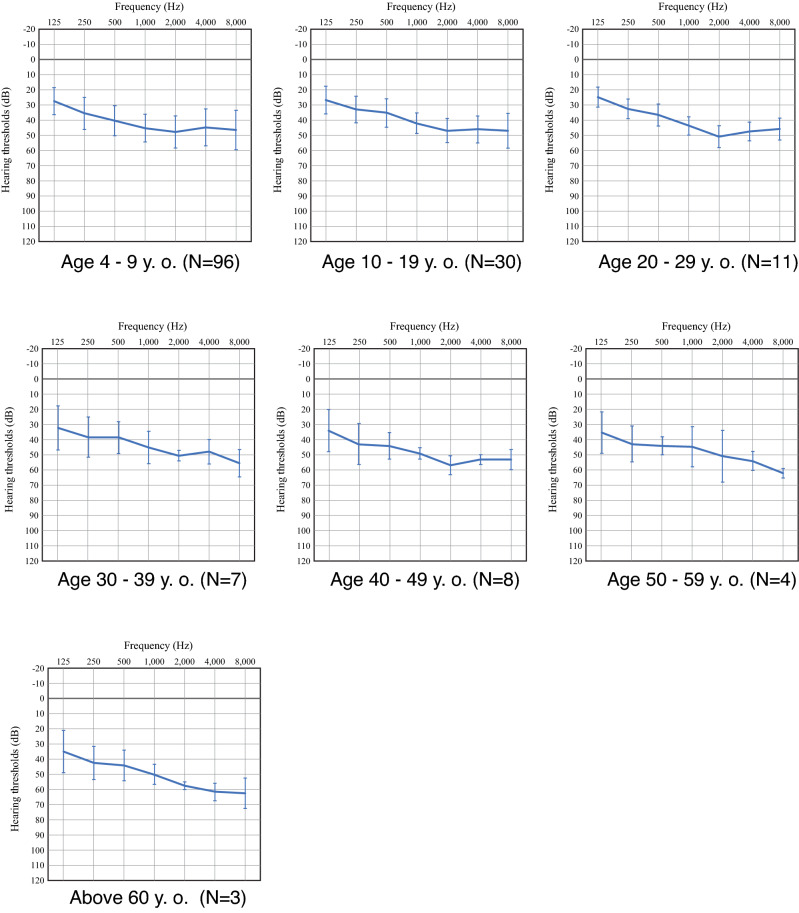
Averaged audiograms of *STRC*-associated hearing loss patients. The hearing threshold data were obtained from 159 probands and show the averaged hearing thresholds with standard deviation for every 10 years of age.

In this study, we collected the hearing thresholds for 7 frequencies (125 Hz, 250H, 500 Hz, 1000 Hz, 2000 Hz, 4000 Hz and 8000 Hz) by pure-tone audiometry for 159 patients with *STRC*-associated hearing loss. Many patients in this study were too young to undergo pure-tone audiometry. We also excluded the patients without hearing thresholds information and two severe-to-profound hearing loss patients carrying both of *GJB2* and *STRC* biallelic mutations. Figure 1 shows the averaged hearing thresholds for every 10 years of age. These results clearly indicate that the characteristic phenotype of *STRC*-associated hearing loss is congenital-onset, non-progressive, mild-to-moderate hearing loss.

### Types and frequencies of *STRC*-*CATSPER2* deletions identified in *STRC*-associated hearing loss patients

**Figure 2:**
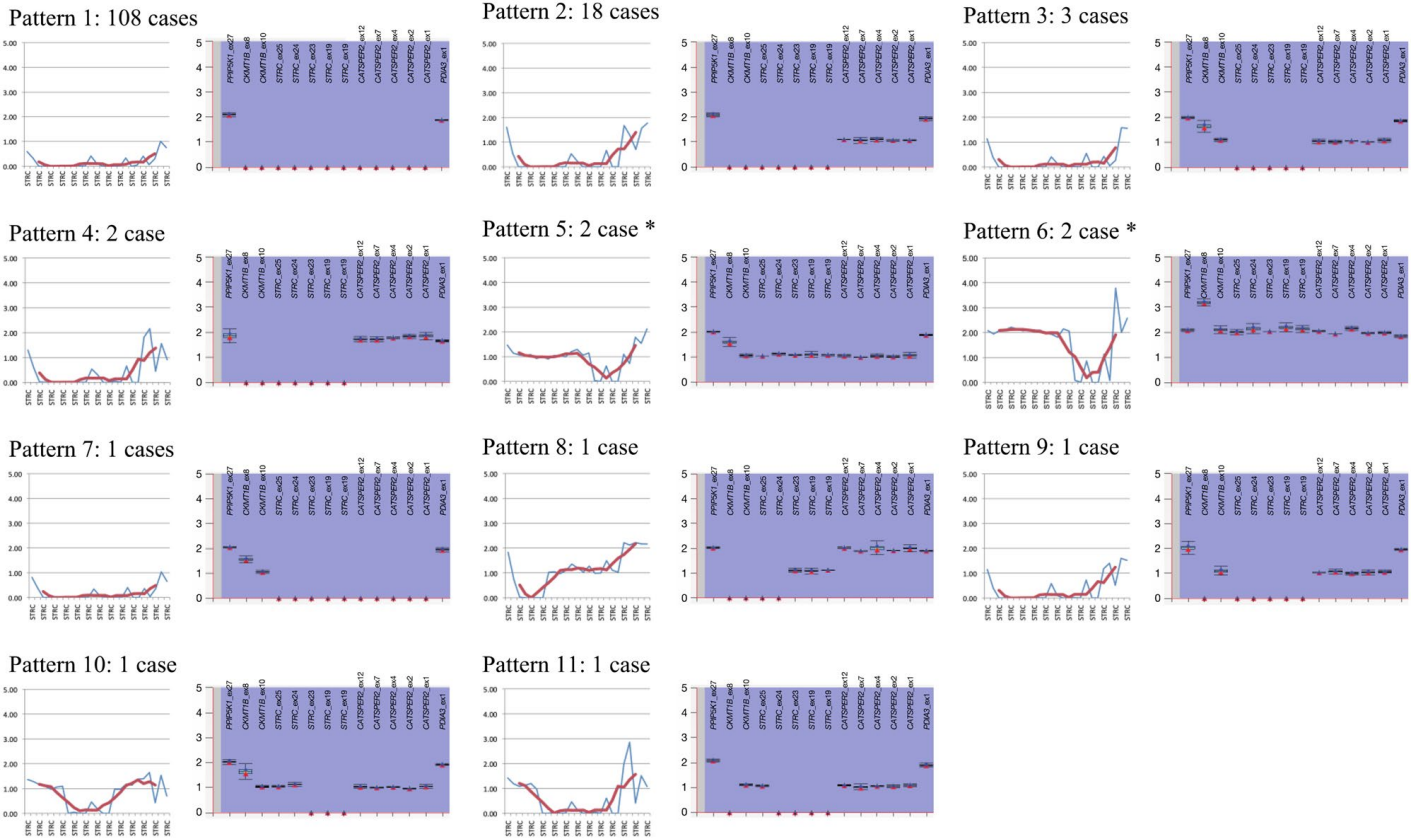
Types of homozygous *STRC* gene deletion identified by MLPA analyses. In this study, we identified 11 types of *CKMT1B-STRC-CATSPER2* gene deletion by NGS and MLPA analyses. NGS-based CNV analysis (left) was performed in accordance with our previous report^22^. Blue indicates the estimated copy number for each amplicon and red indicates the smoothing value for five relative amplicons. The vertical axis shows the copy number for each amplicon. MLPA (right) was performed using a commercially available MLPA probe (SALSA MLPA Probe mix P461-A1; MRC-Holland, Amsterdam, Netherlands). Box plots and error bars indicated the estimated copy number range and standard deviation for each MLPA probe, respectively. The vertical axis shows the copy number for each probe and the horizontal axis indicates the probes used in the MLPA analysis. *The MLPA probe set used in this study was only designed for exons 19, 23, 24 and 25 of the *STRC* gene and did not cover exons 1 to 18. Some patients carried CNVs in these exons and we could detect CNVs by NGS data analysis, but not by MLPA (such as Pattern 5 or 6), in these cases.

To confirm the *STRC* gene deletions identified using the NGS data, we performed MLPA analysis for 140 randomly selected patients with a 2 copy number loss and 18 patients with a 1 copy number loss of the *STRC* gene and SNVs using a commercially available MLPA probe set (SALSA MLPA Probemix P461-A1 DIS). As a result of the MLPA analysis, we identified 11 types of deletion pattern for cases with a 2 copy number loss (Fig. 2 and Supplemental Table 1). The most prevalent deletion pattern was a 2 copy number loss of the whole *CKMT1B-STRC-CATSPER2* gene region, which was identified in 77.1% of cases (108/140), followed by a 2 copy number loss of the *CKMT1B-STRC* gene region with a 1 copy number loss of the *CATSPER2* gene in 12.9% of cases (18/140). In addition, several complex deletion or duplication patterns were observed in some cases. Among 108 cases with a 2 copy number loss of the whole *CKMT1B-STRC-CATSPER2* gene region, 56 cases were female and 52 cases were male. Thus, the prevalence of DIS in patients with hearing loss associated with a 2 copy number loss of the *STRC* gene was 37.1% (52/140).

## Discussion

In this study, we identified homozygous deletions of the *STRC* gene (a 2 copy number loss) in 231 of 9956 Japanese hearing loss patients (2.32%; 231/9956), and 41 cases with a 1 copy number loss of the *STRC* gene which also carried SNVs in the *trans* allele configuration. We also identified 4 cases with homozygous or compound heterozygous SNVs; thus, the prevalence of *STRC*-associated hearing loss in Japanese hearing loss patients was 2.77% (276/9956). In our previous study, we identified 17 of 1025 (1.7%) Japanese hearing loss patients carrying homozygous *STRC* deletions^9^. Based on micro-droplet digital PCR analysis, Ito et al. reported that 2 of 84 (2.4%) Japanese mild-to-moderate hearing loss patients carried a homozygous *STRC* deletion^10^. Similarly, the prevalence of *STRC*-associated hearing loss has been reported to range from 5.4 to 16.1% in hearing loss patients of mixed ethnicity, and in American and Italian populations^4–8^, 2.6% in Turkish autosomal recessive hearing loss patients^11^, and 10% and 10.8%, respectively, in Czech and Korean mild-to-moderate hearing loss patients^12,13^. From these reports, *STRC*-associated hearing loss is concluded to be a relatively common cause of hearing loss in every ethnic population. We previously reported the carrier frequency of the *STRC* deletion in Japanese controls with normal hearing to be 2.6% (4/152)^9^. Ito et al. reported the carrier frequency of the *STRC* deletion in Japanese controls with normal hearing to be 0.9% of (1/107)^10^, whereas Hoppman et al. estimated the carrier frequency of the *STRC* deletion in controls with normal hearing to be 1%^23^.

In the present study, we showed that most *STRC*-associated hearing loss patients exhibited mild-to-moderate hearing loss that did not progress up to the age of 60 (Fig. 1). This type of hearing loss is consistent with that described in previous studies^9,10,13,14,23–25^.

We also identified 11 types of deletion pattern for the homozygous *STRC* gene deletion (Fig. 2), with the most prevalent deletion pattern being the homozygous deletion of the whole *CKMT1B-STRC-CATSPER2* gene region, which was identified in 77.1% of cases (108/140). Thus, most of the homozygous *STRC* deletions associated hearing loss cases carried a 2 copy number loss of the whole *CKMT1B-STRC-CATSPER2* gene region. Among 108 cases with a 2 copy loss of the whole *CKMT1B-STRC-CATSPER2* gene region, 52 cases were male and the prevalence of DIS in patients with hearing loss associated with a 2 copy number loss of the *STRC* gene was estimated to be 37.1% (52/140). Unfortunately, the main symptom of our study cohort was hearing loss and the data were collected from otolaryngology departments; thus, only limited information was obtained regarding male infertility. The phenotype-genotype correlation between our MLPA results and male infertility, therefore, remains unclear. However, a non-negligible portion of patients with homozygous *STRC* gene deletions carried the intact *CATSPER2* gene. Thus, the determination of the deletion range for *STRC*-associated hearing loss patients should be performed to allow more appropriate genetic counseling in terms of hearing loss and male infertility. It is noteworthy that the commercially available MLPA probes for DIS are designed for only exons 19 to 25 of the *STRC* gene and did not identify a partial deletion of exons 1 to 18 of the *STRC* gene. Indeed, we could not identify *STRC* gene deletions for 4 cases with partial deletions of the *STRC* gene (Fig. 2, deletion pattern number 5 and 6). Thus, NGS-based analysis is more powerful and appropriate for the identification of *STRC* deletions.

In conclusion, we herein report that the prevalence of *STRC*-associated hearing loss was 2.77% in a large cohort of Japanese hearing loss patients. In addition, 77.6% of cases (108/140) with a homozygous *STRC* deletion carried a two copy number loss of the whole *CKMT1B-STRC-CATSPER2* gene region. This information will be useful in allowing more appropriate genetic counseling regarding hearing loss and male infertility for patients with *STRC* deletions.

## Supplementary Information

Supplementary Information.
